# Draft Genome Sequences of Two Pseudomonas aeruginosa Isolates from the Female Urogenital Tract

**DOI:** 10.1128/MRA.01378-19

**Published:** 2020-01-02

**Authors:** Genevieve Johnson, Carine R. Mores, Alan J. Wolfe, Catherine Putonti

**Affiliations:** a Department of Microbiology and Immunology, Stritch School of Medicine, Loyola University Chicago, Maywood, Illinois, USA; b Department of Biology, Loyola University Chicago, Chicago, Illinois, USA; c Bioinformatics Program, Loyola University Chicago, Chicago, Illinois, USA; d Department of Computer Science, Loyola University Chicago, Chicago, Illinois, USA; University of Arizona

## Abstract

Pseudomonas aeruginosa is a Gram-negative bacterium that has the ability to survive in and readily adapt to a variety of environmental conditions. Here, we report 2 genome sequences of P. aeruginosa strains, UMB1046 and UMB5686, isolated from the female urogenital tract.

## ANNOUNCEMENT

Pseudomonas aeruginosa is an opportunistic pathogen in compromised hosts but is harmless to healthy individuals. P. aeruginosa is associated with chronic lung infections in individuals with cystic fibrosis (1), as well as nosocomial urinary tract infections (2). While it is not frequently found within the urogenital microbiota of healthy women (3, 4), strains have been isolated from women with lower urinary tract symptoms (3, 5, 6). Here, we present the genomes of two P. aeruginosa strains isolated from two different women. P. aeruginosa UMB5686 was isolated from a vaginal swab sample obtained from a woman with overactive bladder (OAB) symptoms after 12 weeks of treatment with a vaginal estrogen cream (5). P. aeruginosa UMB1046 was isolated from a catheterized urine sample obtained from a woman with a urinary tract infection (6).

P. aeruginosa UMB1046 and UMB5686 were isolated from prior institutional review board (IRB)-approved studies (5, 6) using the expanded quantitative urinary culture (EQUC) protocol (5). Briefly, vaginal swabs were collected using the BD liquid Amies elution swab (ESwab) collection system and cultured as previously described (5); catheterized urine samples were cultured as previously described (6). The genus and species for these isolates were determined via matrix-assisted laser desorption ionization–time of flight (MALDI-TOF) mass spectrometry prior to storage at –80°C. From these freezer stocks, each P. aeruginosa isolate was first streaked on an LB agar plate and incubated at 37°C for 24 hours. A single colony was selected from each plate to inoculate LB broth and incubated at 37°C with shaking for 24 hours. DNA was extracted using the Qiagen DNeasy UltraClean microbial kit and quantified using the Qubit fluorometer. DNA was sent to the Microbial Genomic Sequencing Center (MiGS) at the University of Pittsburgh for sequencing, where the DNA was first enzymatically fragmented using an Illumina tagmentation enzyme. Indices were attached using PCR and sequenced using an Illumina NextSeq 500 flow cell, producing 938,702 and 1,641,347 pairs of 151-bp reads for UMB1046 and UMB5686, respectively. Raw reads were trimmed using Sickle v1.33 (https://github.com/najoshi/sickle) and assembled using SPAdes v3.13.0 with the “only-assembler” option for k values of 55, 77, 99, and 127 (7). Genome coverage was calculated using BBMap v38.47 (https://sourceforge.net/projects/bbmap/). The NCBI Prokaryotic Genome Annotation Pipeline (PGAP) v4.8 (8) was used to annotate the genome sequences. Unless previously noted, default parameters were used for each software tool.

The P. aeruginosa UMB1046 genome is 6,513,817 bp long in 161 contigs with a GC content of 64%, genome coverage of 34.03×, and an *N*_50_ score of 64,232 bp. The P. aeruginosa UMB5686 genome has a similar size, 6,684,697 bp, with a GC content of 63%. The UMB5686 assembly includes 98 contigs with a coverage of 58.48× and an *N*_50_ score of 120,243 bp. PGAP annotation identified 6,257 and 6,351 protein-coding genes for UMB1046 and UMB5686, respectively. The strains vary in their number of rRNA operons and tRNAs; UMB1046 carries 3 rRNA operons and 58 tRNAs, whereas UMB5686 carries 4 rRNA operons and 59 tRNAs. Future analyses of these strains and genomes will further our understanding of this opportunistic pathogen within the female urogenital tract.

### Data availability.

This whole-genome shotgun (WGS) project has been deposited in GenBank under the accession numbers WHVN00000000 and WHVM00000000 for P. aeruginosa UMB1046 and UMB5686, respectively. The raw sequence reads have been deposited under accession numbers SRR10336114 and SRR10336113 for P. aeruginosa UMB1046 and UMB5686, respectively. The WGS and SRA records are associated with BioProject number PRJNA316969.
